# Does anomie help to explain social participation in very old age? A mediation analysis

**DOI:** 10.1007/s10433-026-00926-9

**Published:** 2026-06-22

**Authors:** Luise Geithner, Michael Wagner

**Affiliations:** 1 Cologne Center of Ethics, Rights, Economics, and Social Sciences of Health (Ceres), University of Cologne, Cologne, Germany; 2 Department of Sociology and Social Psychology (DSS), University of Cologne, Cologne, Germany

**Keywords:** Adults aged 80 and above, Social participation, Anomie, Population-based survey

## Abstract

Social participation in old age is beneficial for the individual and the community. However, very old adults are confronted with specific challenges regarding their resources, values, and social networks. In a rapidly changing society, very old adults may experience conflicts resulting in individual experiences of anomie which we understand as feelings of disorientation. They can have negative effects on their social participation. Using data from the population-based survey Old Age in Germany (D80+), we examined whether individual experiences of anomie played a mediating role in explaining very old adults’ social participation. Based on the civic voluntarism model and theories of anomie and ageing, hypotheses were derived and tested using a structural equation model (SEM). Predictors included in the model were socio-economic and health resources, individual values, and social relationships. The SEM revealed significant indirect effects through anomie for education, income, and health. Direct effects on social participation were significant for all predictors except income. While mediation through anomie played a minor role for education and health, its importance for income was greater. Furthermore, model results showed a significant direct effect of all predictors on anomie and of anomie on social participation. While the predictors of social participation according to the civic voluntarism model could be confirmed for very old age, individual experiences of anomie seemed to play a crucial mediating role only for income. The model indicated that not income itself, but rather the insecurities caused by relative deprivation, influenced the social participation of very old adults.

**Supplementary Information:**

The online version contains supplementary material available at 10.1007/s10433-026-00926-9.

## Introduction

Being an active part of a community with reliable social contacts and social exchange is considered an important factor in ageing well and successfully (Rowe and Kahn 1997; Douglas et al. 2017). Social participation in old age has been shown to be associated with better health (Douglas et al. 2017; Chiao et al. 2011), greater cognitive abilities (Bourassa et al. 2017; James et al. 2011; Vogel and Zank 2025), and higher overall quality of life (Douglas et al. 2017; Townsend et al. 2021; Vogel and Zank 2025; Fernández-Mayoralas et al. 2015). Furthermore, formal (e.g. volunteering) and informal (e.g. family assistance) social engagement among older people is expected to have benefits for the community (Simonson et al. 2024; Serrat et al. 2020). However, maintaining social participation can be challenging for very old people. Studies have shown that the number and the frequency of social activities decline with age, particularly from age 80 onwards (Agahi et al. 2006; Pinto and Neri 2017; Finkel et al. 2018; Galenkamp et al. 2016).

Withdrawal from social activities in old age is an ambivalent topic in social gerontology. On the one hand, the view that withdrawal is a natural process that is useful for the very old person and for society is, for example, supported by disengagement theory (Cumming and Henry 1961). On the other hand, withdrawal has been characterised as a sign of entry into the so-called “fourth age” (Higgs and Gilleard 2014; Baltes and Smith 2003), which is typically associated with frailty, dependency on care, and reduced autonomy. The concept of active ageing (Lessenich 2015; World Health Organization 2002; Havighurst 1961) is a popular paradigm promoted by social policy aimed at helping older people stay healthy, (formally) involved, and productive (World Health Organization 2002; Lessenich 2015).

However, it is unclear how very old adults themselves feel about their social involvement and the expectations placed on them, especially when they are confronted with different challenges. The risk of losing individual resources, and especially health resources, is high in very old age (Rott and Jopp 2012; Ophey et al. 2023). Moreover, very old adults’ attitudes about themselves and the world may shift towards placing greater importance on social and emotional values (Jopp et al. 2013; Borg et al. 2017; Vilar et al. 2020). Furthermore, social networks become smaller and more focused on strong relationships associated with positive emotions (Wrzus et al. 2013; Cornwell and Schafer 2016; Böger and Huxhold 2018; Ellwardt and Hank 2023). Consequently, very old adults may experience imbalances between social expectations that they remain active and productive and the opportunities and wishes they actually have.

Such imbalances can cause individual experiences of anomie (or anomia), which refers to feelings of disorientation and insecurity (Heitmeyer 2007; Legge et al. 2008; Legge and Heitmeyer 2012). Anomie has rarely been studied in the context of social participation. Individual responses to experiences of anomie can be diverse, with crime and delinquency (Messner et al. 2008), discrimination and authoritarianism (Hövermann et al. 2015b; Heitmeyer 2024), extremism (Nickel and Groß 2023; Ionescu et al. 2021), and suicide (Durkheim 2022) being the most studied ones. However, another possible response to anomie is “retreatism” (Merton 1968, 1938) or social withdrawal (Bohle et al. 2015). Against this background, the aim of the paper is to examine the mediating role of anomie in explaining social participation in very old age. We investigate the mediating role of anomie by testing theory-driven hypotheses and using a population-based survey of adults aged 80 years and over in Germany (Old Age in Germany–D80+).

### Social participation and influencing factors in old age

The concept of social participation is used in different ways. Terms like social activities, social engagement, or social involvement are often used interchangeably. Although there is no standard definition of social participation, it has, for example, been defined as “a person’s involvement in activities that provide social interactions with others in society or the community” (Levasseur et al. 2010). Six levels of participation can be differentiated based on the level of involvement and the goals of the activity: (1) doing something alone in preparation for connecting with others; (2) doing something when others are around; (3) being in social contact with others; (4) collaborating with others to perform an activity; (5) helping others; and (6) contributing to society, including by engaging in civic activities. While all six levels can be understood as forms of participation, levels 3–6 can be characterised as forms of social participation and levels 5 and 6 as forms of social engagement (Levasseur et al. 2010).

A well-known model for explaining the differing degrees to which people are socially engaged is the civic voluntarism model (Verba et al. 1995). According to this model, the influencing factors are: (1) the person’s resources, such as time, money, or skills; (2) the person’s motives, interests, and beliefs; and (3) the presence of people in the person’s social network who ask for participation (Erlinghagen and Hank 2023). A systematic literature review by Townsend et al. (2021) has shown that these three factors—resources, motives, and social relations—play a crucial role in explaining the social participation of older adults. There is strong evidence that financial resources like income, cultural resources like education, and health resources like functional disability can serve as barriers or facilitators to social participation in later life. Moreover, individual motivations like prosocial motives, altruism, and intrapersonal motivators are associated with the social participation of older adults. Features of the individual’s social network, including their kin and non-kin relationships, the cohesiveness of their relationships, or the duration of residing in a community, have been found to be significant barriers or facilitators to social participation in old age.

### Challenges to social participation in very old age

Very old adults are confronted with age-related changes in the above-mentioned predictors for social participation.

*Resources:* As socio-economic and health resources are associated with social recognition and status, people try to accumulate or keep these resources throughout their life course (Bourdieu 2017). However, physiological and neuropsychological ageing processes increase the probability of developing diseases and experiencing declines in physical and cognitive abilities (Brijoux et al. 2021; Ophey et al. 2023; Hajek et al. 2023). This has consequences for resources bound to the body, namely incorporated cultural capital (e.g. skills), body capital (e.g. stature), and health capital (Bourdieu 2017, 1992; Reeves 2016). Furthermore, cultural capital may become outdated due to social change or lack of usage, and educational degrees and occupational qualifications obtained a long time ago can lose their value (Bourdieu 2014; Ecarius 1996; Müller 1992; Prahl and Schroeter 1996). People’s economic capital may be consumed by living costs and, for those with long-term care needs, by the rising costs of professional nursing care. This decline in resources can become particularly challenging for very old adults with low income, increasing their poverty risk (Geyer 2015; OECD 2024).

*Motives:* Motivational orientations are strongly affected by individual values (Borg et al. 2017). Theories and studies on life span development show that individual values, although relatively stable, are adjusted to the different conditions across the life course (Vilar et al. 2020). While younger people tend to place more emphasis on values promoting personal goals like personal achievement, older people often place greater importance on values promoting social goals like belonging (Borg et al. 2017; Milfont et al. 2016; Gouveia et al. 2015). It is assumed that for people at the end of their life, supra-personal values, emotion-related goals (Vilar et al. 2020), and generativity values (Erikson 1998) gain in importance as they perceive their remaining time as limited (Carstensen 2006). Many of the studies on individual values use Schwartz’s theory of basic human values (Schwartz 1992, 2012). The model contrasts values aiming for openness to change with values aiming for conservation, and values aiming for self-enhancement with values striving for self-transcendence. Studies have shown that the importance of values aiming for self-transcendence (universalism, benevolence) and conservation (tradition, security, conformity) increases with age, while the importance of values reflecting self-enhancement (power, achievement) and openness to change (self-direction, hedonism, stimulation) decreases with age (Borg 2021; Borg et al. 2017; Reissmann et al. 2021b; Robinson 2013; Vilar et al. 2020).

*Social relationships:* Older adults are confronted with an increased risk of having a shrinking social network, usually due to a deterioration in health or critical life events like widowhood or relocation (Wrzus et al. 2013; Cornwell and Schafer 2016). However, social network theory and a number of studies have suggested that for older adults, even small social networks can be satisfying and functional (Ellwardt and Hank 2023), provided they are heterogeneous and contain family members and friends. Especially family roles (e.g. being a partner or a parent) and friend roles are perceived by older adults as very important (Vidovićová 2018). While such social roles entail duties and responsibilities, they can also offer security and orientation (Prahl and Schroeter 1996). These roles are of increasing importance to older adults, as life after retirement is not marked by further institutional transitions providing a planning horizon (Motel-Klingebiel et al. 2013). Nevertheless, for very old people with greater health impairments and long-term care needs it becomes increasingly difficult to maintain or build up new social relationships. Age-associated negative stereotypes (Reissmann et al. 2021a) and ageism (Kessler and Warner 2023) can exacerbate these difficulties.

All in all, experiencing these age-related changes, while also facing a rapidly changing society, can make the social participation of very old adults challenging.

### The explanatory role of anomie

According to the institutional anomie theory (Messner et al. 2008), today’s societies are increasingly characterised by market-based principles like utility, efficiency, competition, and accumulation of money. Such a “marketised mentality” (Bieliński and Hövermann 2023; Hövermann et al. 2016) imposes risks on people who seem to be unable to perform according to these principles, such as people with disabilities, who are at risk of devaluation (Hövermann et al. 2015a, 2015b; Hövermann and Messner 2023).

The perception of such imbalances can cause individual experiences of anomie. Anomie of an individual (or anomia) is understood as an imbalance between the individual’s aims and expectations for social integration and their chances or available means to realise them (Bohle et al. 2015). Durkheim (2022) and Merton (Merton 1968, 1938) have explained how an anomic society, which is characterised by a reduced regulatory power of societal institutions, a reduced bonding force of norms, and a dominance of economic principles, can cause individual experiences of anomie, like a loss of orientation, goals of action, and control, or, more generally, feelings of insecurity about how to act (Hüpping 2007; Mansel et al. 2007; Reuband 2023; Legge et al. 2008).

Based on Durkheim and Merton, Heitmeyer (2015, 2016, 2018, 2024) has further developed the theory to explain group-focused enmity. They posit that anomie is caused by rapid social change and modernisation. This can, in turn, cause imbalances in three different modes of social integration: socio-structural, institutional, and socio-emotional (Bohle et al. 2015; Mansel et al. 2007; Heitmeyer 2024). Imbalances in the three modes of social integration can occur for the following reasons:

- social inequality, social exclusion, or the endangerment of one’s social status (socio-structural);
- declining adherence to basic values and norms (institutional); and
- dissolution and weakening of social ties (socio-emotional).

The modes of social integration allow for participation, a feeling of belonging, and social recognition. Reduced or endangered chances for integration can be individually perceived as a loss of positional, moral, and emotional recognition. Hence, individuals experiencing such imbalances may develop anomie. Consequences can emerge at a cognitive level (e.g. identity problems) and at a behavioural level (e.g. suicide, rebellion, and withdrawal) (Bohle et al. 2015; Endrikat et al. 2016; Hüpping 2007; Mansel et al. 2007).

### The present study

**Fig. 1:**
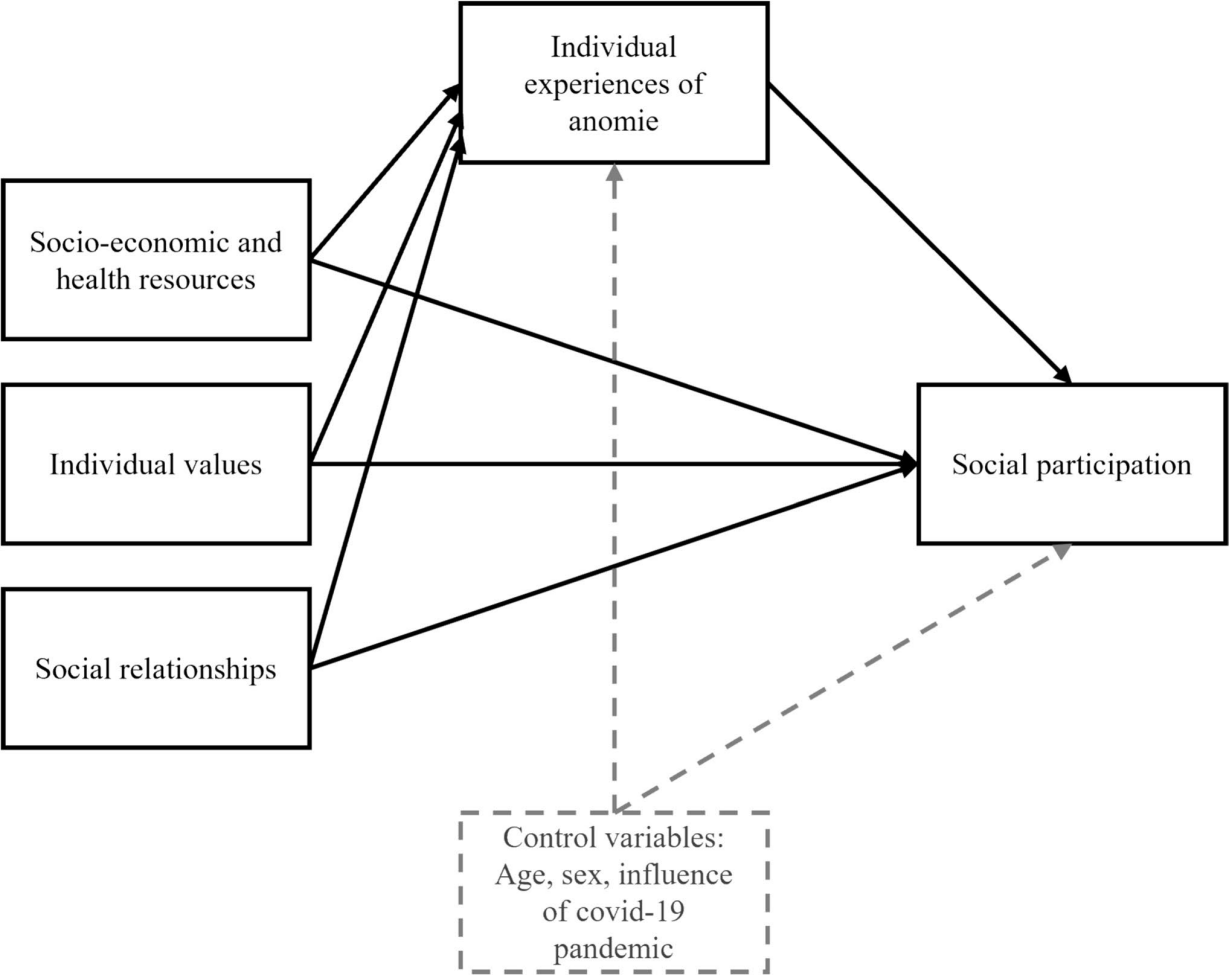
Mediation model showing direct and indirect relations with social participation

Against this background, the present study addresses the mediating role of anomie in explaining social participation in very old age. We pay attention to the mediating role of anomie in the three predictors of social participation: (1) resources, (2) motives (as reflected by individual values), and (3) social relationships (Fig. 1). Based on the above-mentioned theories, we assume that very old adults perceive anomie-causing imbalances due to the age-related changes in these three predictors, which, in turn, cause reduced social participation. We examine the mediating role of anomie through a three-step procedure based on the following hypotheses:

#### H1

Very old adults report stronger experiences of anomie if they have:

- fewer socio-economic and health resources;
- individual values aiming for self-transcendence; or
- fewer social relationships.

#### H2

Very old adults who report stronger experiences of anomie have lower levels of social participation.

#### H3

Experiences of anomie mediate the relationship between very old adults’ social participation and their socio-economic and health resources, individual values, and social relationships.

## Methods and research design

### Data source and study sample

Our analyses are based on the population-based study “Old Age in Germany–D80+ ” (Stuth 2023; Albrecht et al. 2022), which includes data from 10,578 people aged 80 and over and was conducted from November 2020 to October 2021 by the Cologne Center for Ethics, Rights, Economics, and Social Sciences of Health (ceres) in cooperation with the German Centre of Gerontology (DZA). This study was the first systematic survey of very old people in Germany. It was realised by implementing a sampling plan without limits on higher ages that included people living in institutional care settings, and with proxy interviews being conducted for study members who were not able to participate in person because of health impairments. The respondents were asked about a variety of topics covering, for example, their living and health conditions, their experiences with the Covid-19 pandemic, and their social relations. Their cognitive performance was also assessed. Due to the Covid-19 pandemic, the survey was divided into two modules: Essential information was collected by questionnaires sent to the homes of the target persons (module 1), and more complex information was collected by telephone six months later (module 2).^1^

### Measures

#### Outcome: social participation

Social participation was measured as a mean score of three variables capturing the frequency of social activities and the frequency of instrumental and emotional social support given to others. With reference to the above-mentioned definition by Levasseur et al. (2010), the very old adults’ social participation in this study encompasses social contact, collaboration with others to perform an activity, and help for others. The respondents’ answers to the following questions were added up to generate a score, with higher values indicating higher social participation: (1) “In your free time, how often do you spend time with other people (relatives, acquaintances, or friends), exchange ideas, or do something together?” (2) “In the past twelve months, how often have you helped other people with their tasks or errands?” (3) “How often have you comforted other persons or cheered them up?” Answers were given on a five-point response scale from 1 (never) to 5 (very often/always).

#### Mediator: anomie

Anomie was included in the model as a continuous latent variable, with higher values indicating stronger anomic experiences. Three observed variables asking for the respondents’ felt discrepancy with today’s society were used for estimation. The three variables captured the feeling of having a diverging lifestyle, having dissonant values, and experiencing rapid social change. The following three questions were asked: (1) “Do you have the feeling that you are coping less and less with today’s social way of life?” (2) “Do you have the feeling that your own values are becoming less and less compatible with the values of today’s society?” (3) “Do you have the feeling that today’s society is changing so quickly that you no longer know how to orient yourself in society?” Response options ranged from 1 (not at all) to 4 (very much). This anomie scale was developed with reference to Gümüs et al. (2004), Fischer and Kohr (2014), and Brandtstädter et al. (1997). It measures anomie unidimensionally with a focus on the underlying experience of disorientation and insecurity which is in line with the measurement used by Heitmeyer (2007). It allows a focus on the perceived difficulties of the very old population in adapting to changing normative standards, differentiating between right and wrong, and finding stable points of reference in an increasingly complex society. The concept of anomie distinguishes itself from the broader concept of alienation which often includes further dimensions, for example social isolation and powerlessness (Seeman 1959; Lytkina 2020), or from the concept of (non-)belonging with its focus on place, for example place attachment (Oswald et al. 2010; Chaudhury and Oswald 2019). With a Cronbach’s alpha of 0.82, internal consistency of the anomie scale was found to be high in D80+ .

#### Predictor 1: socio-economic and health resources

To measure the very old adults’ socio-economic resources, variables indicating their education and income were used. To measure education, the participants were asked about their highest educational degree and professional qualification. It was coded according to the International Standard Classification of Education (ISCED), which resulted in nine ordinal categories ranging from 0 (early childhood education) to 8 (second stage of tertiary education/doctoral or equivalent) (OECD, European Union, UNESCO-UIS 2015).

Income was measured by the personal monthly equivalised disposable income, which is the income after tax and other deductions adjusted for household size and composition. For D80+ , it was calculated by dividing the total monthly net household income by the equivalised household size. The first person of the household (the respondent) received the weight 1.0, and every other person in the household received the weight 0.5 (Eurostat 2021). For participants living in a nursing home, a one-person household was assumed. The variable was categorised into groups ranging from 1 (below €1,000) to 7 (more than €18,000).

Functional health was used to assess the very old adults’ health resources, as it focuses on the effects of health restrictions on the ability to carry out basic activities. Functional health was measured by the ability to perform instrumental activities of daily living (IADL) (Lawton and Brody 1969). Participants reported how much help they needed to perform the following activities: preparing meals, managing medication, doing housework, grocery shopping, using the telephone, managing finances, and organising transportation. Participants were able to choose from the following response scale: not possible without help (0), some help needed (1), and no help needed (2). A mean score ranging from 0 to 2 was calculated, with higher values indicating less help needed and better functional health.

#### Predictor 2: individual values

Individual values were included in the model by a latent variable reflecting self-transcendence. According to Schwartz (1992), values aiming for self-transcendence, like benevolence and universalism, are in opposition to values aiming for self-enhancement, like striving for power/wealth, achievement, and hedonism. The questionnaire in D80 + consisted of a total of 10 questions, with each question capturing one of the 10 dimensions of the Schwartz value model (Reissmann and Wagner 2023). The items are based on the portrait value questionnaire (Schwartz 2003). To investigate its congruence to the four-factor structure proposed by Schwartz (self-enhancement, openness to change, self-transcendence, and conservation), a confirmatory factor analysis was performed. Fit indices indicated a low model fit. Thus, an exploratory factor analysis was conducted to investigate the underlying factor structure. Results showed that the D80+ sample is best represented by a three-factor model (see supplementary information Table 1). Self-enhancement is represented by power, achievement, hedonism, and stimulation, while self-transcendence is represented by universalism, benevolence, and tradition. This is in accordance with prior studies using the NRW80+ sample (Reissmann et al. 2021b). Thus, three items rating the importance of “to do something good for society” (benevolence), “to take care of nature and the environment” (universalism), and “to respect traditions you have learned from your family or religion” (tradition) were used to measure self-transcendence.^2^ Answers were given on a scale ranging from 1 (not at all important) to 4 (very important). The internal consistency was found to be acceptable, with a Cronbach’s alpha of 0.63. Further psychometric testing for the use in other studies is required.

#### Predictor 3: social relationships

Having a spouse or partner, having children, and having friends were included in the model as dichotomous indicators of important social relationships. These three variables were added up and a mean score was calculated, with higher values indicating more social relationships. Information on marital status and current partnership was used to calculate the first variable. For the second variable, participants’ responses to the question “Do you have or have had children and if so, how many?” were used. When interpreting these results, it should be taken into account that answers to this question may have included children who were no longer alive. The information on being a friend was taken from the respondents’ answers about their social network. Respondents were asked about their relationships with up to six persons whom they identified as being the most important to them. We considered the existence of these relationships as an important condition for social participation in very old age. Based on the civic voluntarism model (Verba et al. 1995) and the importance of family and friend roles in later life (Vidovićová 2018), the presence of these people in the person’s social network may encourage social participation, for example, by friends asking for help.

#### Control variables

Age, sex, and the reported influence of the Covid-19 pandemic were used as control variables. Age was included as a continuous variable. To control for the Covid-19 pandemic, three variables were included to capture the influence of the Covid-19 pandemic on (1) alienation from society, (2) everyday life and leisure time, and (3) private contacts. Response options ranged from 1 (not at all) to 5 (very).

## Analysis

To examine our hypotheses, we specified a structural equation model (SEM) using the estimator WLSMV. The Spearman correlation test was used to assess the relationship between variables. Analyses were carried out with Mplus; Spearman correlation tests were done with SPSS. The final analysis was adjusted for the complex sampling design of the D80+ study including clusters. All analyses used sample weights, and 95% confidence intervals were reported. The analyses included the realised proxy interviews.

Missing values were highest for the mean score of social relationships (*n* = 3089), the monthly equivalised disposable income (*n* = 1927), and the mean score of social participation (*n* = 1423). Due to missing data, the sample size was reduced to *n* = 5883 (pairwise deletion). As the missing data amounted to 44%, we decided to impute the data. We used five imputed datasets, as Asparouhov and Muthén (2022) have shown that results do not change much with a higher number of imputed datasets, particularly with the estimator WLSMV. When results with and without multiple imputation were compared (see supplementary information Table 2), the effects were in the same direction, but the significance changed.

The overall logic of the model followed a segmentation approach (Rungtusanatham et al. 2014; Memon et al. 2018) with the three above-mentioned hypotheses being tested: (H1) the direct effect of the predictor variables on anomie (a-path), (H2) the direct effect of anomie on social participation (b-path), and (H3) the indirect effect of the predictor variables on social participation (ab-path). To estimate how much of the total effect of each predictor variable was mediated by anomie, we calculated the proportion mediated as the quotient of the indirect effect and the total effect.

The following indices were used to assess model fit: Chi-square value *x*^2^ and degrees of freedom (df), variance explained *R*^2^, root mean square error of approximation (RMSEA; should be smaller than 0.06), and comparative fit index (CFI; should be greater than 0.95) (Hu and Bentler 1999).

## Results

### Descriptive statistics

Characteristics of the analysis sample (total *n* = 10,578; *n* = 41 proxy participants) are reported in Table 1.

**Table 1:** Descriptive characteristics of the sample

Variable		N	Mean (SD)	Per cent
Sex	Male	4012		37.9
	Female	6566		62.1
Age		10,578	85.5 (4.13)	
Socio-economic and health resources				
Education (ISCED 0–8)		10,239	3.6 (1.75)	
	Low (0–2)	2415		23.6
	Medium (3–4)	5279		51.6
	High (5–8)	2545		24.9
Monthly equivalised disposable income		9313	1873.6 (1206.36)	
IADL score (0–2)		10,450	1.4 (0.66)	
Individual values: Self-transcendence				
Doing good for society	Not (at all) important	3231		30.5
	(Rather) very important	6782		67.7
Taking care of nature	Not (at all) important	1680		16.5
	(Rather) very important	8473		83.5
Respect traditions	Not (at all) important	1,573		15.4
	(Rather) very important	8657		84.6
Social relationships				
Social relationship score (0–1)		9968	0.2 (0.20)	
Partner status	No partner	5406		53.9
	With partner	4630		46.1
Children	No children	1038		9.9
	Min. 1 child	9445		90.1
Friends	No friends	5546		69.8
	Min. 1 friend	2399		30.2
Individual experiences of anomie				
Way of life	(Rather) not true	4805		47.9
	(Rather) true	5222		52.1
Values	(Rather) not true	3160		31.4
	(Rather) true	6895		68.6
Orientation	(Rather) not true	4473		44.6
	(Rather) true	5555		55.4
Social participation				
Social participation score (1–5)		9858	3.1 (0.78)	
Spending time with others	Never or rarely	2248		22.1
	Sometimes	3797		37.3
	(Very) often	4135		40.6
Given instrumental support	Never or rarely	5855		60.8
	Sometimes	2495		25.9
	Often or always	1280		13.3
Given emotional support	Never or rarely	3959		39.7
	Sometimes	4032		40.4
	Often or always	1981		19.9

Statistics are based on weighted data

About 40% of the very old adults reported spending time with others often or very often, but a much smaller share reported having given emotional (19.9%) or instrumental support (13.3%) to others. Individual experiences of anomie were found to be widespread in the very old population in Germany. About 52% of the participants reported having difficulties with today’s social way of life, about 55% said they no longer know what to look for, and almost 69% said they have the feeling that their own values do not fit the values of today’s society. Given that the study D80+ was conducted during the Covid-19 pandemic,^3^ we compared anomic experiences and social participation with the results of the preceding study NRW80+ (see Table 3 supplementary information), which used the same measurement instrument. We found that the participants of the D80+ study reported having greater difficulties coping with today’s social way of life and with orienting themselves in today’s society. Furthermore, the values for the frequency of spending time with others and the exchange of instrumental and emotional support were lower in the D80+ study.

### Measurement model

Before the hypothesised relationships were examined, the measurement model was assessed (without multiple imputation, *n* = 10,377; WLSMV). The two latent constructs of self-transcendence and anomie, each measured by three manifest variables, were included in the model. Loadings of the manifest variables on the respective latent constructs were statistically significant (*p* < 0.001) and strong, ranging from *B* = 0.760 to *B* = 0.902 for anomie and from *B* = 0.529 to *B* = 0.724 for self-transcendence (standardised). Variance explained (*R*^2^) ranges from 0.578 (orientation) to 0.814 (way of life) for anomie and from 0.280 (traditions) to 0.550 (nature) for self-transcendence. The measurement model showed a satisfactory fit: *x*^2^ = 387.465, df = 8, *p* < 0.001; RMSEA = 0.068, CFI = 0.987. The latent constructs of self-transcendence and anomie were significantly correlated (*B* = − 0.056, *p* < 0.001).

### Mediation analysis

Before the mediation analysis was performed, bivariate correlations were examined (Table 2). The highest bivariate correlations were found for the items measuring anomie.

**Table 2:** Spearman correlation coefficients, means and standard deviation (SD) between the observed study variables (*n* = 10,578)

	Education	Income	Health	Society	Nature	Tradition	Partner	Child	Friend	Sex	Age	Daily life	Contacts	Alienation	Way of life	Values	Orientation	Time	Help	Consolation
Education	1.00																			
Income	0.38**	1.00																		
Health	0.22**	0.14**	1.00																	
Values: Society	0.11**	0.08**	0.18**	1.00																
Values: Nature	0.14**	0.06**	0.29**	0.44^**^	1.00															
Values: Tradition	− 0.04**	− 0.06**	0.04**	0.31^**^	0.32^**^	1.00														
Partner	0.25**	− 0.09**	0.24**	0.08^**^	0.14^**^	0.01	1.00													
Child	− 0.05**	− 0.04**	− 0.04**	− 0.02*	− 0.02*	− 0.00	0.06^**^	1.00												
Friend	0.16**	0.15**	0.19**	0.13^**^	0.15^**^	0.04^**^	− 0.04^**^	− 0.13^**^	1.00											
Sex	− 0.36**	− 0.12**	− 0.21**	− 0.03^**^	− 0.07^**^	0.09^**^	− 0.44^**^	− 0.01	0.08^**^	1.00										
Age	− 0.15**	− 0.03**	− 0.40^**^	− 0.10^**^	− 0.16^**^	0.01	− 0.29^**^	− 0.02^*^	− 0.14^**^	0.12^**^	1.00									
Covid-19: Daily life	− 0.00	0.03**	− 0.03^**^	0.07^**^	0.04^**^	0.05^**^	− 0.05^**^	− 0.01	0.09^**^	0.10^**^	− 0.02*	1.00								
Covid-19: Contacts	− 0.02*	0.03*	− 0.11^**^	0.02^**^	0.00	0.07^**^	− 0.02	0.03^**^	0.06^**^	0.09^**^	0.01	0.51^**^	1.00							
Covid-19: Alienation	− 0.08**	− 0.08**	− 0.19^**^	− 0.01	− 0.04^**^	0.06^**^	− 0.04^**^	0.01	− 0.03^*^	0.07^**^	0.03^**^	0.38^**^	0.43^**^	1.00						
Anomie: Way of life	− 0.11**	− 0.10**	− 0.26^**^	− 0.09^**^	− 0.04^**^	0.05^**^	− 0.07^**^	− 0.01	− 0.08^**^	0.07^**^	0.12^**^	0.08^**^	0.14^**^	0.28^**^	1.00					
Anomie: Values	− 0.05**	− 0.05**	− 0.13^**^	− 0.03^*^	0.02	0.10^**^	− 0.03^**^	− 0.00	− 0.05^**^	0.05^**^	0.09^**^	0.07^**^	0.11^**^	0.19^**^	0.65^**^	1.00				
Anomie: Orientation	− 0.19**	− 0.17**	− 0.30^**^	− 0.08^**^	− 0.07^**^	0.07^**^	− 0.11^**^	0.02	− 0.13^**^	0.09^**^	0.15^**^	0.05^**^	0.10^**^	0.28^**^	0.61^**^	0.54^**^	1.00			
Participation: Time	0.05**	0.06**	0.22^**^	0.17^**^	0.12^**^	0.10^**^	0.01	0.07^**^	0.12^**^	0.06^**^	− 0.09^**^	0.03^**^	0.00	− 0.09^**^	− 0.13^**^	− 0.10^**^	− 0.13^**^	1.00		
Participation: Help	0.20**	0.10**	0.34^**^	0.24^**^	0.21^**^	0.07^**^	0.20^**^	0.00	0.14^**^	− 0.18^**^	− 0.22^**^	0.06^**^	− 0.01	− 0.06^**^	− 0.12^**^	− 0.05^**^	− 0.17^**^	0.23^**^	1.00	
Participation: Consolation	0.08**	0.05**	0.13^**^	0.28^**^	0.22^**^	0.19^**^	0.03^**^	0.02	0.18^**^	0.08^**^	− 0.09^**^	0.12^**^	0.09^**^	0.04**	− 0.03^**^	0.03^*^	− 0.07^**^	0.22^**^	0.41^**^	1.00
Means	3.61	2.92	1.40	2.73	3.13	3.22	0.46	0.90	0.30	1.62	85.5	2.29	2.83	2.10	2.48	2.81	2.59	3.23	2.19	2.65
SD	1.75	1.21	0.66	0.77	0.81	0.80	0.50	0.30	0.46	0.49	4.13	1.25	1.31	1.10	1.0	0.90	1.03	0.95	1.13	1.04

Statistics are based on weighted data. *Correlation is significant at the 0.05 level (2-tailed). **Correlation is significant at the 0.01 level (2-tailed)

**Fig. 2:**
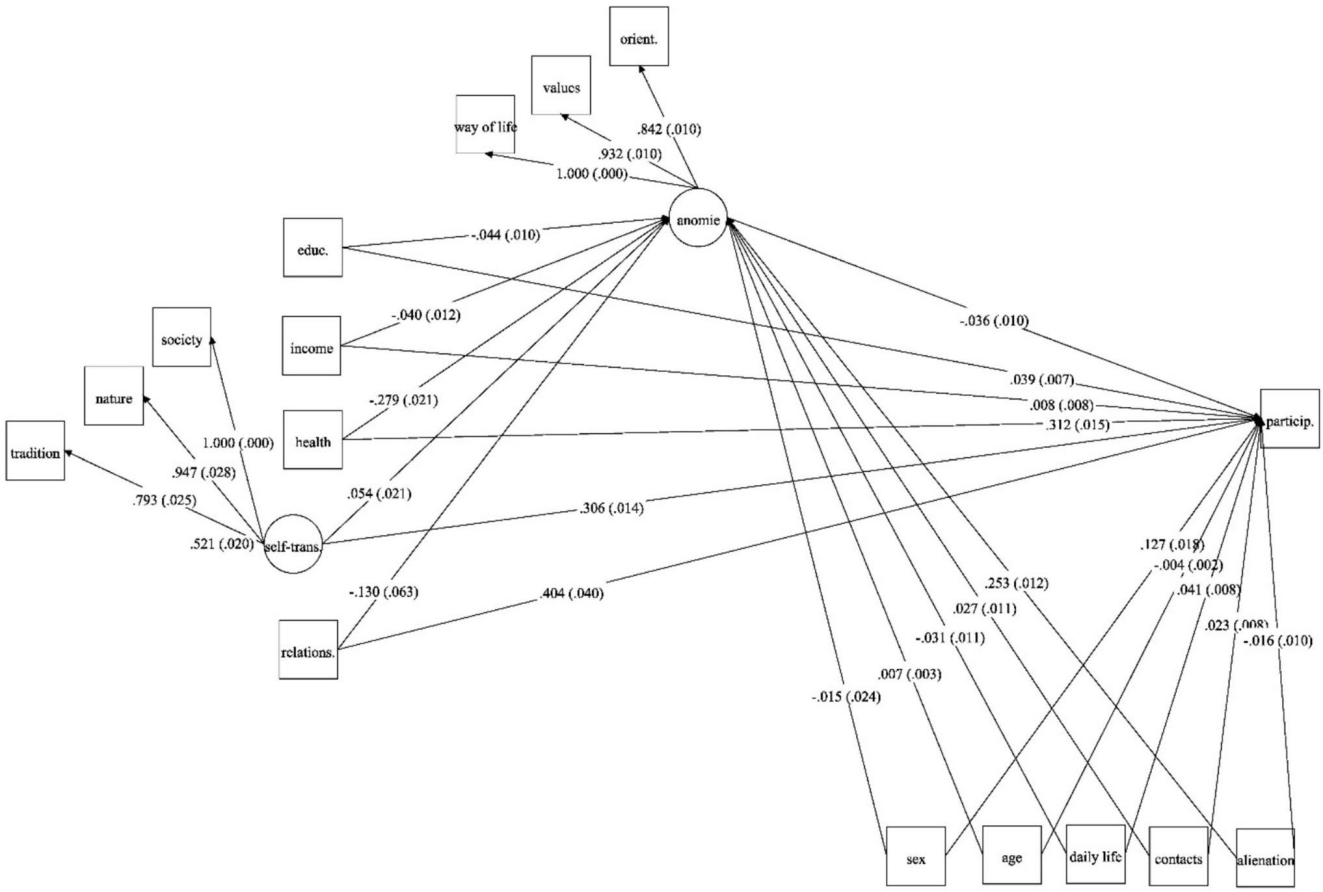
Structural model with unstandardised coefficients and standard errors in parentheses

Results of the mediation analysis are presented in Table 3 for standardised coefficients and in Fig. 2 for unstandardised coefficients. Direct effects on anomie (a-path) were significant for all predictors, which support H1. Moreover, the analysis showed a significant direct effect of anomie on social participation (b-path: B = − 0.046, SE = 0.012, CI = − 0.070–− 0.022, *p* < 0.001), which supports H2. Significant indirect effects through anomie (ab-path) were found in the impact of education, income, and functional health on social participation, which support H3 for these predictors. The percentage of the total effect explained by the mediation was highest for income, at 15.3%. The fit indices confirmed a good fit with *x*^2^ = 1,469.34 (SD = 18.006), df = 57; RMSEA = 0.048 (SD = 0.000), and CFI = 0.949 (SD = 0.001). The results further showed that 18.3% of the variable anomie (*R*^2^ = 0.183) and 23.3% of the variable social participation (*R*^2^ = 0.233) were explained by the model.

**Table 3:** Statistical results of the mediation analysis (*n* = 10,578)

	Direct effect on anomie (a)				Direct effect on participation (c’)				Indirect effect (ab)				Total effect	% mediated
	*B*	SE	95% CI	*p*	*B*	SE	95% CI	*p*	*B*	SE	95% CI	*p*	*B*	
Education	− 0.062	0.014	0.010–0.071	< 0.001	0.070	0.013	0.045–0.094	< 0.001	0.003	0.001	0.001–0.005	0.021	0.073***	0.039 (3.9%)
Income	− 0.051	0.015	− 0.091–− 0.034	0.001	0.013	0.013	− 0.013–0.039	0.345	0.002	0.001	0.001–0.004	0.032	0.015	0.153 (15.3%)
Health	− 0.192	0.014	− 0.219–− 0.164	< 0.001	0.276	0.013	0.251–0.301	< 0.001	0.009	0.003	0.004–0.014	0.007	0.285***	0.031 (3.1%)
Self-transcendence	0.040	0.016	0.010–0.071	0.009	0.295	0.012	0.271–0.318	< 0.001	− 0.002	0.001	− 0.004–0.000	0.267	0.293***	–
Social relationships	− 0.032	0.015	− 0.062–− 0.002	0.039	0.128	0.013	0.103–0.153	< 0.001	0.001	0.001	0.000–0.003	0.123	0.130***	–

Statistics are based on weighted data. Analysis adjusted for sex, age, and influence of the Covid-19 pandemic. *B* = standardised coefficient. SE = standard error. CI = confidence interval. Significance levels: *** *p* < 0.001, ** *p* < 0.01, * *p* < 0.05

## Discussion

Using the civic voluntarism model, we identified several possible predictors of social participation in very old age. In line with our theoretical assumptions, we found that individual resources (education and health), motives/values (self-transcendence), and social relationships (friends, children, partner) were associated with the very old adults’ levels of social participation. To shed more light on the underlying mechanisms, we integrated the concept of anomie. The theory of anomie predicts that individual experiences of disorientation and insecurity can have consequences for people’s behaviour, causing for example “retreatism” (Merton 1968, 1938) or social withdrawal (Bohle et al. 2015). We assumed that very old adults are likely to experience anomie, as they are often confronted with age-related losses of socio-economic and health resources and social relationships, and with a shift in the importance of individual values. This can make it increasingly difficult for them to orient themselves in a society characterised by rapid societal change and the domination of economy-driven principles. Therefore, we investigated whether individual experiences of anomie have a mediating role in explaining social participation in very old age.

The model results showed that anomie, although it is of relevance, has a minor role in mediating the effects on social participation. The indirect effects for education, income, and health on social participation were significant but small. However, the mediating role of anomie in the relationship between the very old adults’ social participation and their income seems to explain a considerable part of the association. This result suggests that material disadvantage operates not only through access to opportunities but also through perceived insecurities and disorientation. These insecurities may evolve as very old age is increasingly described as a time in life with financial risks. Especially in Germany, political discourse largely revolves around insecure public retirement payments, growing costs for long-term care, and rising housing costs. Furthermore, research on anomie has shown that people with lower economic status are not only less satisfied with their opportunities to live their life according to their own wishes and demands, but also that they are more fearful of social exclusion and of the future (Mansel et al. 2007; Hüpping 2007; Heyder and Gaßner 2012) which can be described as relative deprivation (Rippl and Baier 2005). These fears could lead people to withdraw from social activities. Social expectations to age actively and successfully (Havighurst 1961; Rowe and Kahn 1997) may not be met if older adults perceive their future financial situation as uncertain.

The study results must be interpreted with caution, as they are based on cross-sectional data collected during the Covid-19 pandemic. Further research should investigate the explanatory role of anomie using longitudinal data. Based on such data, changes in anomie and the mediating role of anomie could be examined. Our descriptive results have shown that individual experiences of anomie are of considerable relevance in very old age, as a large proportion of the very old population in Germany reports feeling disoriented in today’s society. The reasons and consequences of this feeling of disorientation have been largely unexplored. In this context, attention should be paid to the marginalisation of very old age as an undesirable “fourth age” (Higgs and Gilleard 2014) which stands in contrast to a modern “marketised mentality” (Bieliński and Hövermann 2023; Hövermann et al. 2016) and social expectations to age actively, productively, and successfully. Comparing the results for the very old population with those for other age groups may indicate whether the role of anomie changes with age. Another possible explanation that requires more attention is related to cohort effects. Today’s very old cohorts have experienced specific historical events and were socialised during a specific historical time. This could explain why they feel in tension with today’s dominant values and ways of life.

Although the mediating role of individual experiences of anomie within this study was limited, we were able to show that such perceptions of insecurity and disorientation are of high relevance in later life. Moreover, with the concept of anomie we were able to move beyond the individual level and point to possible societal imbalances which can be described as anomic societies (Durkheim 2022; Merton 1968, 1938). Since very old people, in most cases, do not actively participate in the workforce and often undergo age-related changes in their resources, values, and social networks, they could be particularly affected by the manifestations of economic dominance which become apparent in the devaluation, accommodation, and penetration of non-economic roles and realms of social life (Messner et al. 2008). The emerging socio-structural, institutional, and socio-emotional imbalances (Bohle et al. 2015; Mansel et al. 2007; Heitmeyer 2024) can endanger the social participation of very old people.

## Supplementary Information

Below is the link to the electronic supplementary material.

Supplementary file1 (DOCX 24 KB)

## Data Availability

Access to the scientific use files of D80+ is permitted by the Research Data Centre of the German Centre of Gerontology (FDZ-DZA) at [https://www.dza.de/en/research/fdz/d80/data-access] (https:/www.dza.de/en/research/fdz/d80/data-access).

## Footnotes

1 The study D80 + was based on a preceding regional study in North-Rhine Westfalia, Germany. A population-based study and a follow-up study on the “Quality of life and subjective wellbeing of very old people in North-Rhine Westfalia – NRW80 + ” were conducted between 2017 and 2020 (Kaspar et al. 2024; Wagner et al. 2018). For NRW80 + , existing measurement instruments were analyzed and pre-tested in interviews with very old people regarding their suitability (e.g. by think-aloud). Instruments were adapted where necessary. After that, they were piloted in two rounds by researchers and trained interviewers. Documentation of the methodological preparatory work can be found at the GESIS Leibniz Institute for the Social Sciences (Zank et al. 2022).

2 Although the value aiming for tradition is not a part of the self-transcendence dimension in the Schwartz value model, it is very close to the items of this dimension, which indicates a congruency.

3 The D80+ study contained direct questions about the influence of the Covid-19 pandemic on different areas of life. For example, 13.5% of the very old adults reported that the pandemic had alienated them strongly or very strongly from society (for another 15.2% moderately), 21.2% reported that the pandemic had a strong or very strong influence on their everyday life and leisure (18.4% moderate), and 36.7% said it had a strong or very strong influence on their private contacts (20.3% moderate).
