# Supplementary material for: Does anomie help to explain social participation in very old age? A mediation analysis
Source: Eur J Ageing. 2026 Jun 22;23(1):28. doi: 10.1007/s10433-026-00926-9 (PMC13287186; doi:10.1007/s10433-026-00926-9)
Supplement: Supplementary file 1 — Supplementary file1 (DOCX 24 KB) [file 10433_2026_926_MOESM1_ESM.docx]

**Supplementary information**

**Title:** Does anomie help to explain social participation in very old age? A mediation analysis

**Journal:** European Journal of Ageing

**Authors:** Luise Geithner, Michael Wagner (Universität zu Köln, Department of Sociology and Social Psychology (DSS), Cologne, Germany, mwagner@wiso.uni-koeln.de)

Table 1: Results of the exploratory factor analysis for three factors (n=10,348)

| Variable | Factor loadings | | | Variance |
| --- | --- | --- | --- | --- |
|  | **Factor 1** | **Factor 2** | **Factor 3** |  |
| 1: Self-direction | 0.224* | **0.280*** | 0.087* | 0.794 |
| 2: Power | **0.609*** | -0.010* | -0.378* | 0.705 |
| 3: Security | 0.007 | **0.815*** | -0.002 | 0.333 |
| 4: Hedonism | **0.635*** | 0.067* | -0.137* | 0.635 |
| 5: Benevolence | 0.186* | -0.018* | **0.646*** | 0.442 |
| 6: Achievement | **0.484*** | 0.062* | 0.070* | 0.704 |
| 7: Stimulation | **0.746*** | -0.464* | 0.003* | 0.439 |
| 8: Conformity | 0.106* | **0.237*** | 0.184* | 0.841 |
| 9: Universalism | 0.009 | 0.062* | **0.708*** | 0.464 |
| 10: Tradition | -0.009 | 0.276* | **0.473*** | 0.634 |
| Eigenvalues | 2.867 | 1.575 | 1.164 |  |
| Correlation between factors | | | | |
| Factor 1 | - |  |  |  |
| Factor 2 | 0.307* | - |  |  |
| Factor 3 | 0.473* | 0.275* | **-** |  |

Notes: Statistics are based on weighted data. Geomin rotated loadings. Highest factor loading of variable in bold type. Significance levels: * p<0.05.
Fit indices: RMSEA=0.042, CFI=0.972, SRMR=0.027. Eigenvalues for factors four to ten <1.
For more information on the variables used in the D80+-study see https://www.dza.de/en/research/fdz/d80/documentation.

Table 2: Statistical results of the mediation analysis without multiple imputation (n=5,331)

|  | Direct effect on anomie (a) | | | | Direct effect on participation (c’) | | | | Indirect effect (ab) | | | | Total effect | % mediated |
| --- | --- | --- | --- | --- | --- | --- | --- | --- | --- | --- | --- | --- | --- | --- |
|  | *B* | SE | 95% CI | *p* | *B* | SE | 95% CI | *p* | *B* | SE | 95% CI | *p* | *B* |  |
| Education | -0.076 | 0.018 | -0.111–  -0.040 | **<0.001** | 0.068 | 0.016 | 0.036– 0.100 | **<0.001** | 0.004 | 0.002 | 0.000–0.007 | **0.038** | 0.071*** | 0.050 (5.0%) |
| Income | -0.048 | 0.018 | -0.082– -0.013 | **0.006** | 0.025 | 0.016 | -0.007– 0.057 | 0.125 | 0.002 | 0.001 | 0.000–0.005 | 0.061 | 0.027 | - |
| Health | -0.191 | 0.023 | -0.235– -0.146 | **<0.001** | 0.340 | 0.017 | 0.306– 0.373 | **<0.001** | 0.009 | 0.004 | 0.002–0.016 | **0.015** | 0.349*** | 0.026 (2.6%) |
| Self-transcendence | 0.040 | 0.023 | -0.006– 0.086 | 0.086 | 0.290 | 0.016 | 0.259– 0.322 | **<0.001** | -0.002 | 0.001 | -0.005–0.001 | 0.206 | 0.289*** | - |
| Social relationships | -0.041 | 0.019 | -0.078– -0.004 | **0.030** | 0.127 | 0.017 | 0.094– 0.159 | **<0.001** | 0.002 | 0.001 | 0.000–0.004 | 0.099 | 0.128*** | - |

Notes: Statistics are based on weighted data. Analysis adjusted for sex, age, and influence of the Covid-19 pandemic. Analysis with listwise deletion and bootstrapping (95% bias‐corrected bootstrap CI). *B*= standardised coefficient. SE= standard error. CI= confidence interval. Significance levels: *** p<0.001, ** p<0.01, * p<0.05. Direct effect of anomie on participation: B=-0.047, SE=0.018, CI=-0.082–-0.011, p=0.010.

Table 3: Comparison of individual experiences of anomie and social participation among D80+ and NRW80+ study participants

| Variable | NRW80+ 1^st^ study (n=1,863) | D80+  (n=10,578) |
| --- | --- | --- |
|  | 08/2017-02/2018 | 11/2020- 10/2021 |
| Anomie: |  |  |
| Way of life | 39.0 | 52.1 |
| Values | 64.5 | 68.6 |
| Orientation | 49.5 | 55.4 |
| Participation: |  |  |
| Time with others | 58.5 | 40.6 |
| Help others | 18.2 | 13.3 |
| Consolation | 24.6 | 19.9 |

Notes: Statistics are based on weighted data.

The Covid-19 pandemic started in February/March 2020 and reached its peak from September 2020 to May 2021 in Germany. The first NRW80+ study and the follow-up study were finished before the pandemic or before the contact restrictions were introduced.
